# Molecular size and solubility conditions of polysilane macromolecules with different topology

**DOI:** 10.1038/srep35450

**Published:** 2016-10-17

**Authors:** Andraž Mavrič, Artem Badasyan, Mattia Fanetti, Matjaz Valant

**Affiliations:** 1 University of Nova Gorica, Materials Research Laboratory, Vipavska 13, SI-5000 Nova Gorica, Slovenia; 2 University of Electronic Science and Technology of China, Institute of Fundamental and Frontier Sciences, Chengdu 610054, China

## Abstract

Solubility of polysilane macromolecules has so far been a scientific as well as technological problem due to a lack of understanding of their proper molecular size and agglomeration/de-agglomeration conditions. Here we show that, in contrary to previous reports, the polysilane molecules are inherently small enough to be, under right conditions, dissolved. We used a dynamic light scattering and a differential scanning calorimetry to show that even under a dilute regime the polymer molecules are agglomerated at room temperature and undergo de-agglomeration at slightly elevated temperatures of around 40 °C. The de-agglomeration results in formation of stable solutions of the polymer molecules of different topological structure in different organic solvents. We determined the polymer molecular sizes to be around 20 nm, much lower than previously reported. The measured molecular size was confirmed by transmission electron microscope imaging of the individual molecules.

Polysilanes, i.e. polymers containing silicon in the main chain, find use in many technological applications^1^: they were recognized as precursors to SiC ceramics^2^,^3^ and nanoparticles^4^, potential semiconductors^5^, photoconductors^6^,^7^,^8^ and resists in UV lithography^9^,^10^,^11^. The technological value of the polysilanes is overshadowed by strong molecular interactions leading to problems with solubility. The solubility can be slightly tuned by altering a chemical composition of side chains or polymer length. But, it took fifty years after the first report on insoluble and therefore intractable polydiphenylsilanes in ref. 12 to develop polysilanes that are relatively soluble^13^,^14^,^15^. The limited solubility already enabled numerous technological and industrial applications of the polysilanes. However, the recent attempts to apply the polysilanes in nanotechnologies have exposed insufficient understanding of their structural and physico-chemical parameters. Some problems that occurred are: in lithography line-edge roughness and resolution are affected by the polymer agglomeration^16^; a thickness of polysilane-based hard coatings is limited by a polymer molecule/aggregate size^17^; a size of SiC nanoparticles derived from the polycarbosilanes depends on dispersion of these molecules in precursor solutions^4^ etc. All these purely technological problems have a similar origin, insufficient understanding of a true size distribution of the polymer molecules and their dispersion characteristics in different solvents.

According to the Flory-Huggins theory of mixing or the self-consistent field theory^18^,^19^, dispersion characteristics of a polymer solution can be controlled by tuning solution quality. Altering the polysilane solution quality by changing the solvent is difficult, if not impossible, due to the poor solubility of these polymers in many organic solvents^13^. However, there is an alternative; the solution quality can be adjusted by a temperature change (Fig. 1). Once the system is moved from the two-phase region of the phase diagram to a single diluted phase (transition indicated by the red arrow in Fig. 1), the polymer aggregates within the solution decompose into isolated polymer molecules. Many published results on polysilane solubility, can be explained with the Flory-Huggins theory. For instance, in refs 13, 14, 15 the solubility was improved by applying a higher temperature (~500 K) and using polymers with short chain lengths, respectively. Both factors can effectively move the system out of the two-phase region of the phase diagram into the single-phase region (compare the curves for different *N* in Fig. 1). However, these approaches do not represent a practical solution for technological exploitation of the polysilane macromolecules. A systematic improvement of the solubility of the full-size polymer molecules at temperatures well below a boiling point of the solvents is required.

From 1980’s on, many papers have been devoted to synthesis of the polysilanes (see ref. 1 and references therein), but information on their molecular weights has often been controversial, probably due to the presence of agglomeration and precipitation, which are results of strong inter- and intra-molecular attractions and take place even at very low concentrations^15^,^20^. Typically, experimental data for a crude polymer mixture obtained with Wurtz-type synthesis showed trimodal molecular weight distributions roughly in ranges *M*_w_ < 10^3^, 10^3^ < *M*_w_ < 10^5^ and 10^5^ < *M*_w_ < 10^7^ (see, e.g. refs 1 and 21). The fraction with the lowest molecular weight is mainly oligomeric and not interesting as it is just a consequence of an incomplete reaction. Jones *et al*.^21^ explained the appearance of two other polymer fractions as a result of a competition between different reaction mechanisms for termination of the growth. Alternatively, Zeigler *et al*.^22^,^23^ explained it as a result of the phase separation and differences in steric limitations for the access of the reactive monomer to the growing polymer in diluted or precipitated state.

Majority of the results on molecular size have been reported in terms of molecular weights, although this is not a directly measurable quantity. It can be obtained with light scattering and gel chromatographic techniques that measure quantities related to a hydrodynamic diameter. These results are later converted to the molecular weight^18^. The conversion depends on a size scaling regime of the polymer solutions and can lead to a discrepancy between both techniques^24^. In addition, the measurements strongly depend on polymer solubility^15^,^20^,^25^ and the conditions within the mixing phase diagram. Furthermore, these techniques cannot differentiate between the de-agglomerated and agglomerated particles. Altogether it explains the inconsistencies from the literature, such as the wide reported span of polysilane sizes, from 20 nm to 2 μm^1^,^26^.

## Results and Discussion

**Figure 2:**
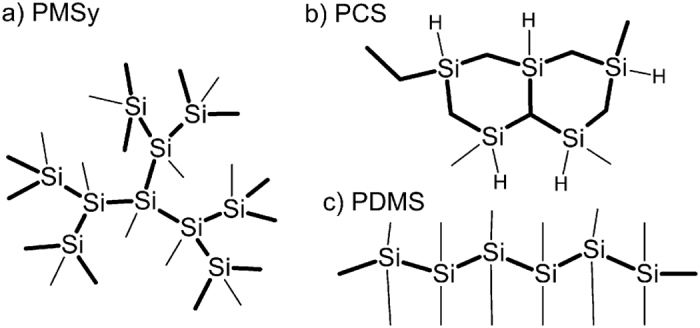
Schematic presentation of structural units of the three polycarbosilane structures with different topologies; (**a**) dendrimer^27^,^28^, (**b**) network^29^ and (**c**) chain^30^,^31^.

Because real molecular sizes of polysilanes are still unknown after almost a century of studies, we focused our research on this problem. Here we present our experimental findings that by increasing temperature just above 40 °C the polysilane agglomerates within a solvent break down into individual molecules. This means that they become soluble. We followed the de-agglomeration process by dynamic light scattering (DLS) and differential scanning calorimetry (DSC) measurements. After full de-agglomeration the molecular size was determined with transmission electron microscope (TEM) imaging. We showed that the de-agglomeration process exhibits very similar characteristics for the polysilanes of different composition and only weakly depends on topology. The understanding of the de-agglomeration process enabled us to experimentally determine the molecular size of three types of polymers with different structure and topology (Fig. 2). The first is polymethylsilyne (PMSy) that is a dendrimer, formed by Si atoms bound to three other Si atoms and a methyl group^27^,^28^. In a polycarbosilane (PCS) layered network the Si atoms are bound to other Si over bridging C atoms^29^. Polydimethylsilane (PDMS) is a linear polymer with Si atoms forming a chain^30^,^31^.

To determine the molecular size of PMSy and PCS we carried out time-dependent DLS measurements of freshly prepared polymer solutions in extra dry tetrahydrofurane (THF). Because PDMS was not soluble in THF in the amount detectable by DLS instrument, the solution was prepared in absolute anhydrous ethanol. Concentrations for all DLS and DSC measurements (2.5 mg/mL for PMSy, 0.05 mg/mL for PDMS and 50 mg/mL for PCS) were kept as low as possible to stay in a dilute regime and just above the detection limit of the instrument.

**Figure 3:**
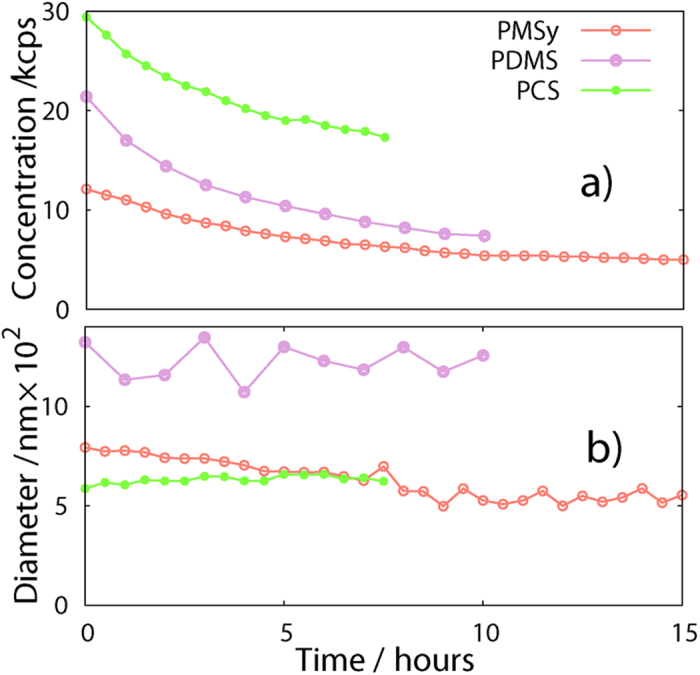
Kinetic DLS measurements of the three polymer solutions: (**a**) concentration (in kcps - kilo counts per second of detected scattering events) decreases with time exponentially; (**b**) hydrodynamic diameter of the particles in the dilute phase is almost constant with time. After some time the signal disappeared due to precipitation.

With analyses performed at 25 °C we detected an occurrence of an exponential process that evolves on the scale of hours. The already low concentration of particles in the dilute solutions decreased below a detection limit of the instrument after several hours (Fig. 3a). During this process the measured average hydrodynamic diameter of the particles remained approximately constant (Fig. 3b). To identify a reason for the decrease in concentration, we treated the samples, for which the DLS signal had already disappeared, with ultrasound. The DLS measurements, performed after the ultrasound treatment, showed that the concentration has recovered back to the initial value. This indicates on a reversible process that cannot involve alternation in covalent bonding. We formulated a working assumption that the solutions are not stable at room temperature and the only possible reason for the observed decrease in concentration is molecular agglomeration. The agglomerates precipitated and segregated on the bottom of a cuvette and thus become invisible to the DLS laser beam. The DLS analysis only gave the signal from the not-yet precipitated particles that remain in the solution. With time their concentration decreased so low that no signal was generated anymore. Additional confirmation came from visual observation. In the initial, clear and transparent solutions we noticed formation of the precipitates; the solutions became cloudy.

**Figure 4:**
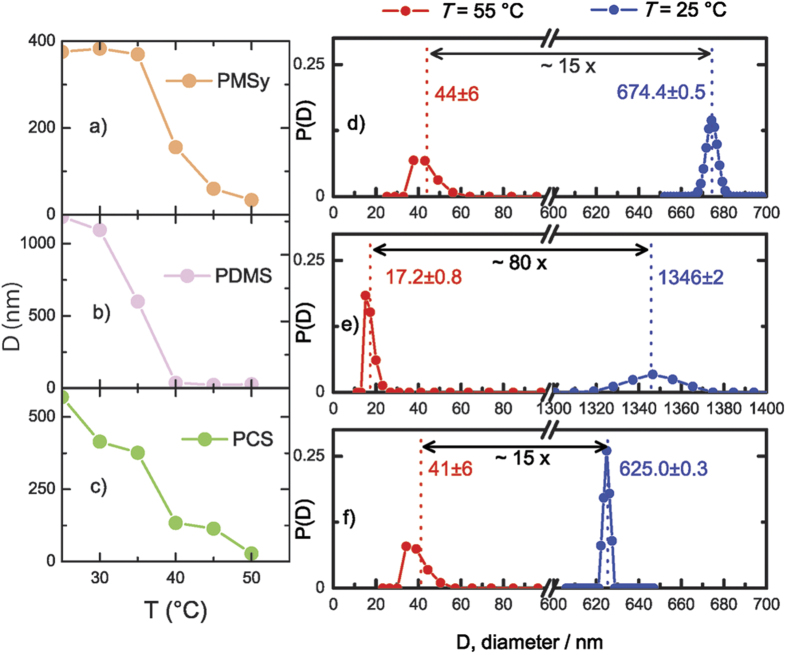
DLS measurements of a particle hydrodynamic diameter, *D*, for the three polymers. Left – temperature dependence of *D* for (**a**) PMSy, (**b**) PDMS and (**c**) PCS. Right - particle size distributions, *P*(*D*), at 25 °C (blue) and 55 °C (red) for (**d**) PMSy, (**e**) PDMS and (**f**) PCS after 5 h of equilibration. Average diameters are shown with numbers and vertical dashed lines. The span of the two-sided black arrows indicates a factor of change of the average diameters, ranging from 15 up to 80 times.

With a goal to prevent the agglomeration we performed a temperature-dependent DLS experiments. The results of the experiments (Fig. 4a–c) show an abrupt drop in the average particle size of all three studied polymers in a temperature range from 25 to 50 °C. The drop is enormous, about two orders of magnitude, from about 1300 nm to as low as 20 nm. The size distribution measurements (Fig. 4d–f) are in full compliance with this.

We consider these results as an indication that the system crossed the binodal line on the Flory-Huggins phase diagram of mixing and appeared in the single-phase regime. To reach the lowest free-energy state, the agglomerates broke down to the individual polymer molecules. The additional support to the idea that we have observed a phase transition (spinodal decomposition) comes from the fact that the DLS curves in Fig. 4 are approximately sigmoidal.

**Figure 5:**
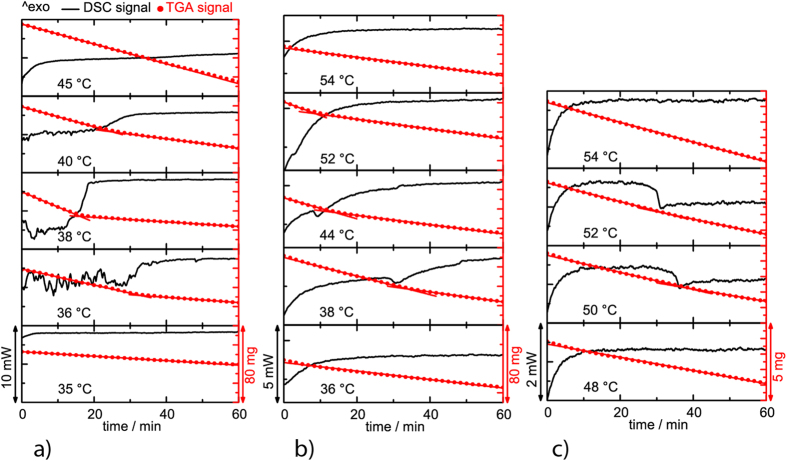
Isothermal DSC and TGA kinetic studies of (**a**) PCS, (**b**) PMSy and (**c**) PDMS, indicating structural changes with temperature. The DSC signal is weaker for PMSy and PDMS due to lower polymer concentration (lower solubility) compared to the PCS solution.

To confirm that the extensive structural reordering is taking place, we performed isothermal DSC and TGA kinetic studies (Fig. 5). The obtained DSC and TGA curves showed that a change in the solution state occurs at similar temperatures as seen by the DLS analyses. For PCS at 35 °C, flat DSC and TGA lines were recorded (the lowest panel of Fig. 5a). However, at a temperature, just one degree higher (36 °C), multiple endothermic peaks appeared in the DSC signal indicating on the agglomeration/de-agglomeration processes typical for a region of metastability. We can attribute such behavior to the spinodal decomposition of agglomerates and transition to the single phase region in the miscibility phase diagram. An additional confirmation for the de-agglomeration process comes from a sudden change in a slope of the TGA curves, which indicates on a decrease in a solvent evaporation rate. The de-agglomeration reduces the evaporation rate because of the increase in a number of polymer particles in the solvent and an increase in the total polymer-solvent interaction surface. The presence of the multiple peaks on the DSC signal is recorded for all temperatures up to 40 °C. At even higher temperature of 45 °C, the flat DSC and TGA lines were recorded again (the top panel of Fig. 5a), indicating an absence of the phase transition and a completely de-agglomerated state. PMSy and PDMS show qualitatively similar behavior (Fig. 5b,c).

**Figure 6:**
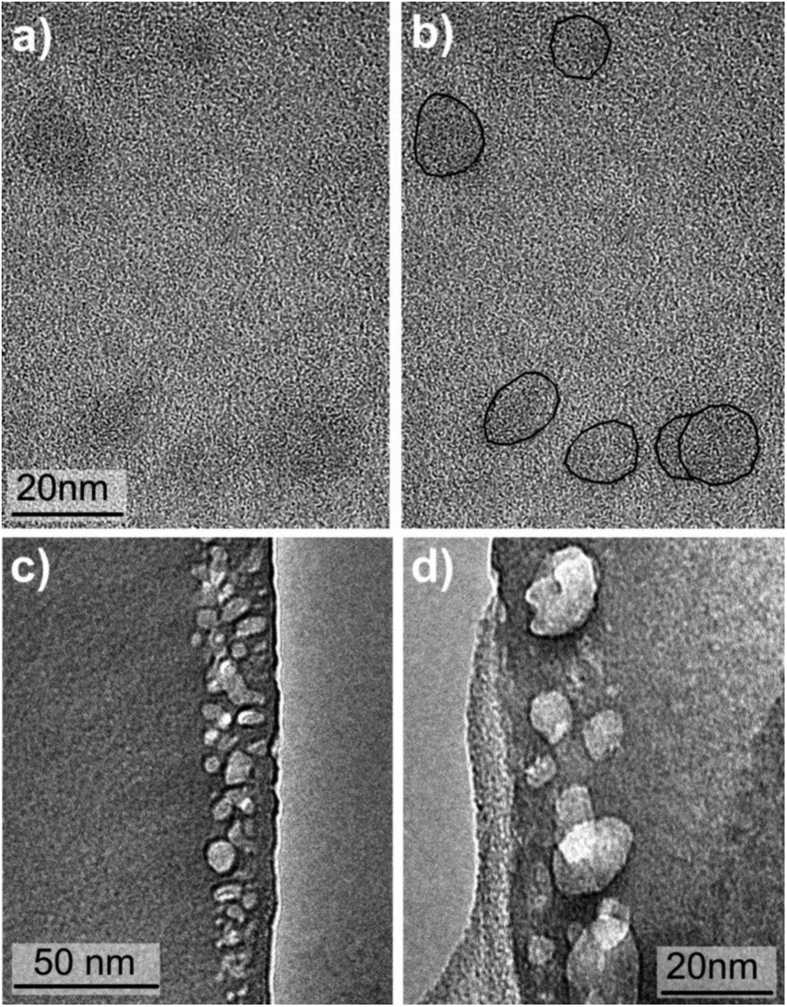
TEM imaging of PMSy molecules; (**a**) individual PMSy molecules deposited directly on a microscopy grid (**b**) the same image with the molecules encircled with the black line for guidance to eyes, (**c**,**d**) dispersed PMSy molecules frozen within a layer of amorphous alumina.

**Figure 7:**
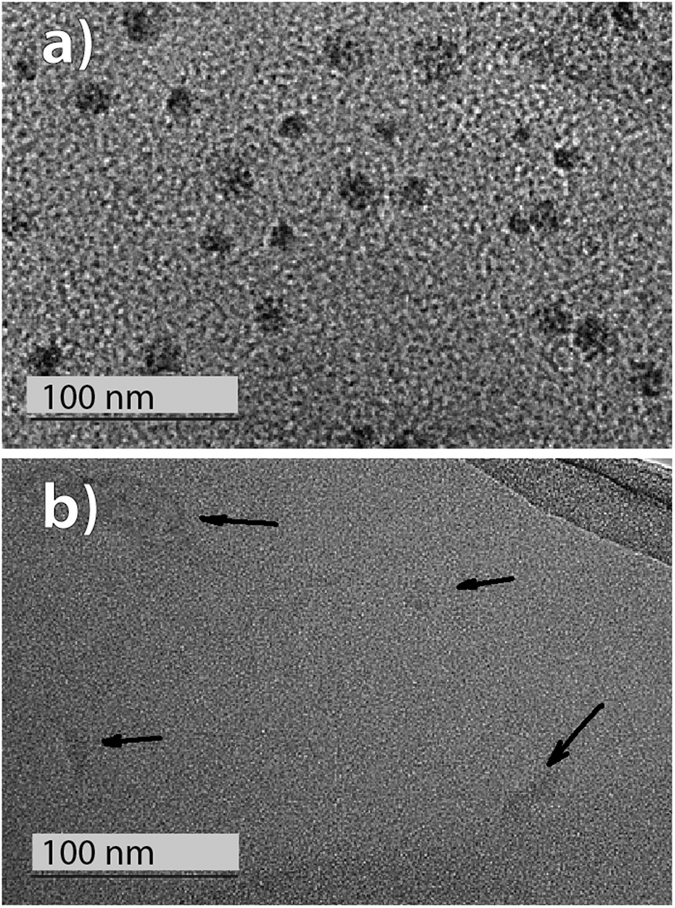
TEM images of individual (**a**) PCS and (**b**) PDMS molecules deposited directly on a microscopy grid. The black arrows indicate the PDMS molecules that are visible only in a low contrast.

While the applied methods used for the size determination of the polysilane molecules gave the hydrodynamic diameters, TEM imaging was used to obtain information on their actual size and shape based on the electron density. The TEM imaging of the polymer molecules is problematic due to a weak electron scattering of the single molecules. The molecules appear as dark globular features about 20 nm in size (Figs 6 and 7), which corresponds to all our previous observations. For even better imaging we developed a new sample preparation procedure. We froze the dispersed polymer molecules within a solid amorphous alumina matrix where any agglomeration is made impossible. The TEM imaging of such system (Fig. 6c,d) with a much better contrast shows the PMSy molecules and their size again to be around 20 nm.

As it was first noticed by De Gennes and Hervet^32^ and later by others^33^,^34^, the volume, occupied by a dendrimer in a space-filling state, grows exponentially with a number of generations, *g*, while the maximum accessible volume only increases cubically with the generations. Since the occupied volume grows faster than the accessible volume, for every dendrimer always exists a maximum attainable generation, *g*_max_, beyond which, due to the sterical reasons, the polymer growth is seriously hindered. At *g*_max_ the dendrimer is in the densest state. Its size (radius of gyration) scales with a number of monomers, *N*, and the monomer size, *b*, according to 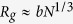. Our theoretical estimate for the radius of gyration, under the assumption that the linkers, connecting the branch points, contain three Si-Si bonds, gives the value of 17 nm, which, translated into a mean particle diameter, using a relationship 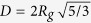, reproduces the experimental value of 44 nm.

To summarize, the key to determine the true molecular size of the polysilanes is in a proper control of solution properties. Due to the agglomeration the molecular size has so far been severely overestimated and the solubility underestimated. To obtain repeatable, time-independent and true information about the polysilane molecular size, the solution must be driven out of the two-phase region of the mixing phase diagram, for instance, by an increase in temperature. At room temperature the polymer molecules are aggregated into large clusters of about micrometer size. These can be de-agglomerated by the increase in temperature to around 40 °C, which results in formation of a stable solution of the polymer molecules in an organic solvent. Within such fully dispersed system the molecular size can be determined in a decisive manner. We have used several different techniques to show that the real molecular size is in the range of around 20 nm. The controversy with previous studies that report much larger hydrodynamic diameters^1^,^24^,^26^,^35^ can be explained with an incomplete de-agglomeration.

Our study also sheds light on the widely discussed trimodal size distribution, appearing in the reaction mixture after the polysilane synthesis^1^,^21^,^22^,^23^. In addition to the oligomeric fraction, the two larger fractions can be explained as a result of the presence of phase separation between the agglomerated and de-agglomerated state.

Through the understanding of the polysilane molecular size and solubility conditions, this study has opened a way for more reproducible and targeted application of the polysilanes in advanced nanotechnology, which so far has not been a case due to misunderstanding of their fundamental structural and chemical parameters.

## Methods

Polymethylsilyne (PMSy) was synthesized using the electrochemical method. The entire synthesis process was conducted in a dry box. In a mixture of 12 mL of acetonitrile (J.T.Baker, Phillipsburg, NJ) and 3 mL of methyltrichlorolsilane monomer, CH_3_SiCl_3_ (Alfa Aesar, Karlsruhe, Germany), 0.5 g of anhydrous AlCl_3_ (Aldrich, Steinheim, Germany) was dissolved. The solution was placed in an electrochemical cell with two aluminum electrodes and left stirring for 15 minutes. A potential of 5.5 V was applied with a power supply PS3020 (HQ Power, Gavere, Belgium) until the current dropped to 99% of the initial current, which assured that the polymerization was completed. The synthesized product was end-capped with 1 mL of 2.0 M LiAlH_4_ in THF (Aldrich, Steinheim, Germany) and left stirring overnight to remove residual chlorine from the polysilane. The product was cleaned with extraction in *n*-pentane (Alfa Aesar, Karlsruhe, Germany) to remove salts. After evaporation of pentane a yellow viscous oil/paste was obtained with a yield of around 50%. PMSy was characterized by ^29^Si solid state NMR. On NMR spectra resonances at the chemical shift typical for PMSy fragments are present, showing Si branching to three, two and one Si atom^36^: −21.5 ppm for -Si(CH_3_)H_2_, −38.2 ppm for = Si(CH_3_)H, −47.5 ppm, −54.0 ppm and −65.1 ppm for ≡ Si(CH_3_). Poly(dimethylsilane) (PDMS) was purchased from Aldrich (Steinheim, Germany) and polycarbosilane (PCS) from Nabond Technologies Co., Ltd. (Hong Kong).

The particle size measurements were preformed with a 90 Plus/BI-MAS DLS system and data were processed with 9KPSDW software package (all Brookhaven Instruments Corporation, Holtsville NY). The solutions were treated in an ultrasound bath for 1 minute to degas and then left 30 minutes at room temperature to equilibrate before DLS measurements. The results were obtained as an average of 6 runs with duration of 5 minute. The temperature dependent particle size is given for 5 °C temperature steps with intermediate 1 h equilibration. The thermal analysis was conducted with a TGA/DSC 2 system (Mettler Toledo) using nitrogen (5.0) as a protective (30 mL/min) and purge (40 mL/min) gas in a 70 μL platinum crucible covered with a lid. The isothermal DSC plots were recorded at 35, 36, 38, 40 and 45 °C for PCS, at 36, 38, 44, 52 and 54 °C for PMSy and at 48, 50, 52 and 54 °C for PDMS (Fig. 5). For TEM observation of single molecules, lacey C-coated Cu TEM grids (SPI, West Chester, PA) were dipped in diluted PMSy (Fig. 6a,b), PCS (Fig. 7a) and PDMS (Fig. 7b) solutions in ethanol and let drying in air. For an alternative method of the TEM sample preparation for the PMSy, the synthesis procedure, described above, was changed so that Al was kept in the system. In the process of purification the solvents were removed under reduced pressure. Water was added to the solid residue in order to deactivate reducing agent and hydrolyze AlCl_3_. The hydrolysis is an important step to prevent Al-loss as a result of AlCl_3_ sublimation. After the centrifugation the excess of water was evaporated and the yellow solid was dissolved in 25 ml of ethanol and filtered or centrifuged again. The stable solution with a molecular dispersed polymer was obtained. The solution was dip-coated on a glass substrate, dried with hot air and heat-treated at 550 °C. The high-resolution TEM microscopy was performed with a transmission electron microscope (JEM 2100F, JEOL) operated at 100 kV and 200 kV (Fig. 6a,b at 100 kV, Figs 6c,d and 7 at 200 KV). The cross-section of the coated substrates was prepared by a standard mechanical sample preparation process and finalized by Ar^+^ ion-milling and polishing (PIPS, GATAN) at grazing incidence (<5°).

## Additional Information

**How to cite this article**: Mavrič, A. *et al*. Molecular size and solubility conditions of polysilane macromolecules with different topology. *Sci. Rep.* **6**, 35450; doi: 10.1038/srep35450 (2016).

## Figures and Tables

**Figure 1:**
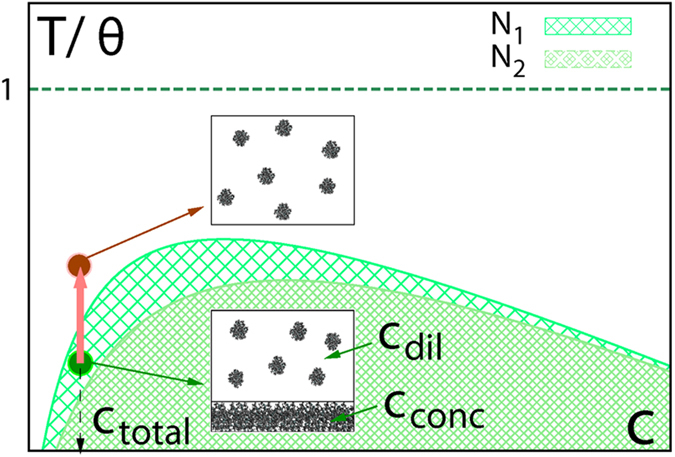
Scheme of a temperature-concentration mixing phase diagram of polymer solutions. Two curves for a different number of repeating polymer units, *N*_1_ > *N*_2_, are shown. Two-phase regions of the phase diagram are shaded. Concentrations of diluted and concentrated phases are determined according to the lever rule. The dashed straight line indicates the temperature, at which excluded volume repulsion is exactly compensated by solvent-mediated attaraction, so-called *θ* temperature.
